# A Case Report on Brachial Plexus Anomaly, Embryological Basis, and Clinical Implications

**DOI:** 10.7759/cureus.49504

**Published:** 2023-11-27

**Authors:** Ashley M Nichols, Dishaben B Patel, Nicole L Geske, William McMillan

**Affiliations:** 1 Radiology, Division of Human Anatomy, Michigan State University College of Osteopathic Medicine, East Lansing, USA; 2 Radiology, Division of Human Anatomy, Michigan State University, East Lansing, USA

**Keywords:** prosection, embryology, human variation, brachial plexus, anatomy

## Abstract

This study presents the routine prosection findings of a 74-year-old male anatomical donor, whose cause of death was attributed to anoxic brain injury secondary to cardiac arrest and acute exacerbation of chronic obstructive pulmonary disease (COPD). The patient exhibited a significant medical history, including severe COPD, chronic heart failure, atrial fibrillation, hypertension, stage III chronic kidney disease, heavy alcohol abuse, obesity, coronary artery disease, peripheral edema, triple bypass surgery, and right hip replacement. A detailed examination of the upper extremities revealed anomalies within the brachial plexus, with a more pronounced presence on the left side. This particular donor deviates from the expected "typical" brachial plexus anatomy, with a lack of convergence into a lateral cord and an anomalous convergence into a superior trunk. To ensure optimal patient care and procedural outcomes, a collaborative approach between surgeons and anesthesiologists that is grounded in a comprehensive understanding of these anatomical nuances is essential. Therefore, this study aims to comprehensively investigate the identified brachial plexus anomalies, elucidate their embryological origins, and explore their clinical implications. Through these objectives, this research contributes to a broader understanding of anatomical variations and their relevance in medical practice.

## Introduction

The brachial plexus has been known to exhibit variation from the typical pattern, with many anomalies that have been previously described [1-4]. The prosected anatomical donor discussed here was found to have an atypical branching pattern not noted in the literature. It is fairly common to have an abnormal fusion of various divisions and branches, especially between the median and musculocutaneous nerves, only one cord, only two trunks, or abnormal branching patterns in the lateral cord [5]. However, there is little literature describing anomalies involving the lack of a superior trunk in addition to the lack of formation of a lateral cord as described here. These observations indicate the presence of variations in the brachial plexus structure arising from embryologic development. The branching pattern of the brachial plexus forms due to physical obstacles around which the nerves must develop. The developmental structures that most frequently influence the branching patterns of the nerves are the arteries and veins, thus variation in the brachial plexus may be explained by variation in the development of vasculature [5]. Given that anatomical anomalies within the brachial plexus are frequently encountered, their potential implications for surgical procedures and anesthesia administration are noteworthy. The aim of this case study was to investigate the embryological explanations for classic and atypical brachial plexus anomalies [5] and consider their implications such as in assessing a shoulder injury, administering a nerve block, or performing neurosurgery or orthopedic surgery in this region [6,7].

The formation of the brachial plexus

The motor and sensory innervation of the upper limb is provided largely by the brachial plexus. The brachial plexus typically receives input from four cervical and one thoracic ventral rami (C5, C6, C7, C8, and T1) [8] and follows the pattern of “roots, trunks, divisions, cords, branches.” The C5 and C6 ventral primary rami merge to form the superior trunk. The C7 ventral primary ramus continues to become the middle trunk. The C8 and T1 ventral primary rami merge to form the inferior trunk. Each trunk then divides into anterior and posterior divisions. The anterior divisions of the superior and middle trunks join to form the lateral cord. The anterior division of the inferior trunk continues to become the medial cord. The posterior divisions of all three trunks join to form the posterior cord. Each cord then further branches to form the five major terminal nerves. The musculocutaneous nerve (formed from C5-6) branches from the lateral cord. The ulnar nerve (formed from C8-T1) branches from the medial cord. The median nerve (formed from C6-T1) receives contributions from both the lateral and medial cords. The axillary nerve (formed from C5-6) and the radial nerve (formed from C5-T1) branch from the posterior cord [9]. Some texts differ slightly in their description of the nerve root contributions to the terminal nerves. For example, some describe the musculocutaneous nerve as being formed from C5-7 nerve roots and the ulnar nerve as often receiving input from the C7 nerve root as well as C8-T1 nerve roots [10,11]. Some texts also list C5 in addition to C6-T1 as contributions to the median nerve [11]. Additional nerves of the brachial plexus include the suprascapular nerve (C5, 6) that branches from the superior trunk, and the lateral and medial pectoral nerves that branch from the lateral and medial cords, respectively [9].

## Case presentation

**Figure 1:**
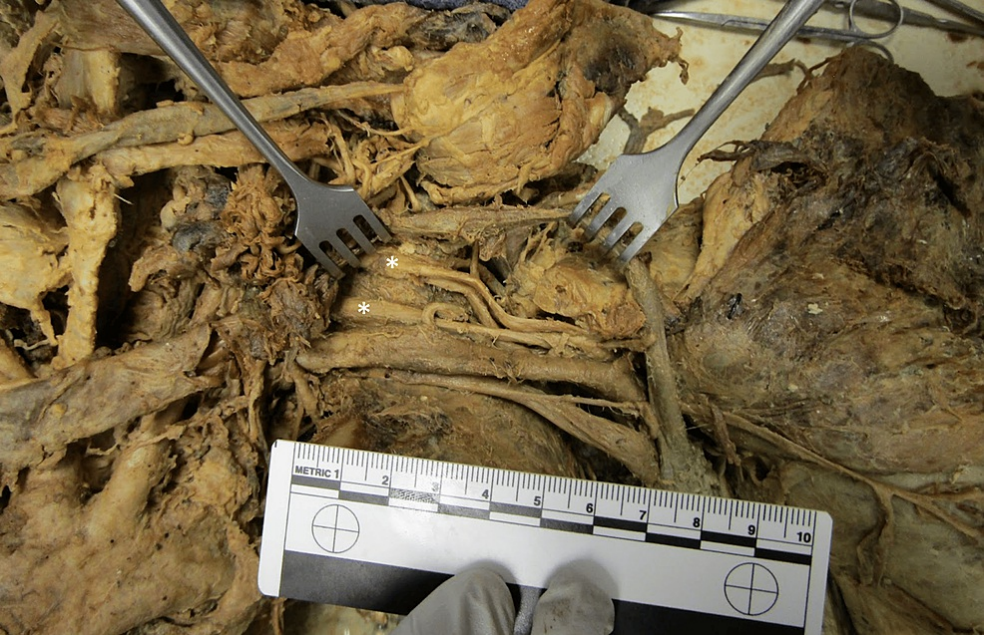
Thick cords of the left brachial plexus Cords marked with an asterisk (*). The ruler in the image measures 10 cm.

During a routine prosection of a 74-year-old male donor, an abnormal right brachial plexus branching pattern was discovered. There was an absence of the superior trunk and lateral cord while the posterior and medial cords exhibited their typical patterns. Additionally, while the left brachial plexus had normal branching patterns, the cords and terminal nerves were thicker when compared to those in the right brachial plexus (Figure 1).

**Figure 2:**
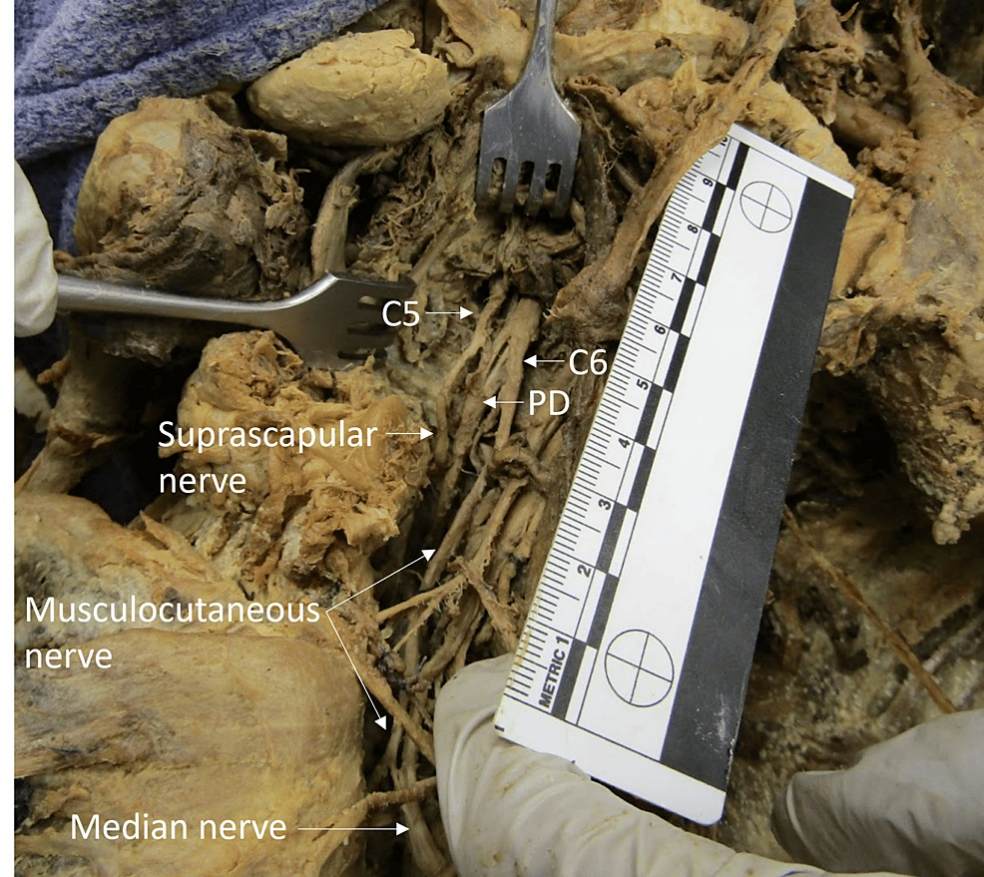
Branching pattern of the C5 and C6 ventral rami The C5 ventral ramus split into an anterior and posterior division without the formation of a superior trunk. The anterior division from the C5 ventral ramus formed the suprascapular nerve. The C6 ventral ramus branched into anterior and posterior divisions. The anterior division continued as the musculocutaneous nerve. The posterior divisions from the C5 and C6 ventral rami (PD) merged. The ruler in the image measures 10 cm.

The right brachial plexus received innervation from the C5-T1 ventral rami, as expected. However, C5 and C6 did not form the superior trunk; instead, both the C5 and C6 ventral rami each split into anterior and posterior divisions. The anterior division of C5 continued as the suprascapular nerve. The anterior division of C6 continued as the musculocutaneous nerve. Typically, the suprascapular and musculocutaneous nerves receive innervation from both C5 and C6. In this individual, only C5 contributed to the suprascapular nerve and only C6 contributed to the musculocutaneous nerve (Figure 2).

The suprascapular nerve branched from the C5 nerve root without the formation of a superior trunk. The C5 nerve root contributed minimally to the brachial plexus. The C6 nerve root then branched into anterior and posterior divisions. The anterior division continued as the musculocutaneous nerve. The ruler in the image measures 10 cm.

**Figure 3:**
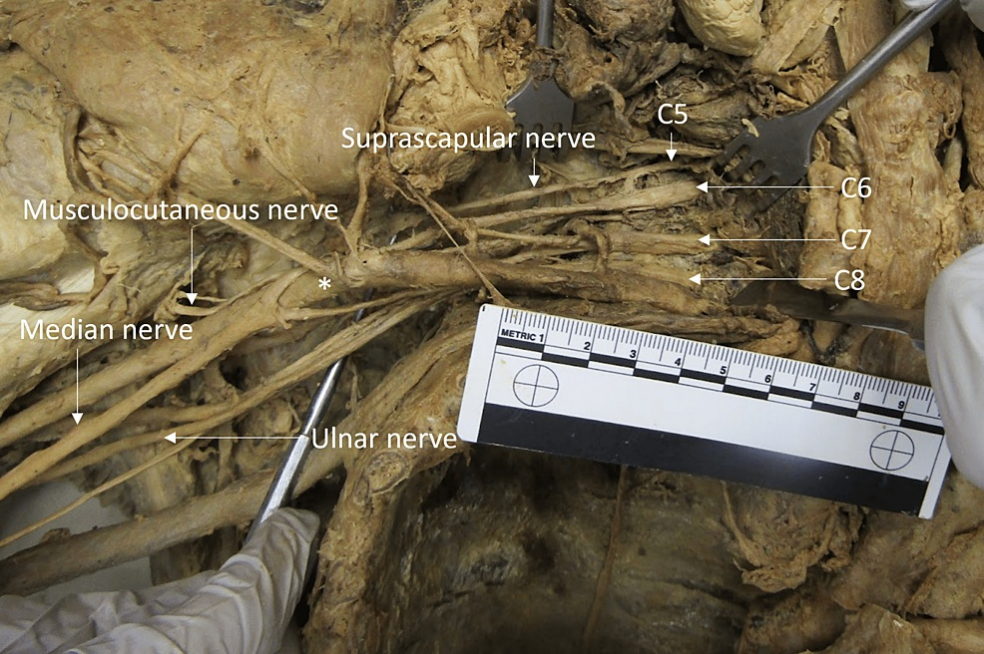
Right brachial plexus median nerve contributions The median nerve received contributions from a branch of the medial cord and two small branches from the anterior division of the C6 ventral ramus. The ruler in the image measures 10 cm.

Typically, the median nerve will receive two branches, one from the lateral cord and and one from the medial cord. In this individual, the median nerve received a branch from the medial cord. However, as there was not a lateral cord, the second contribution came from two very small branches from the anterior division of C6 (the musculocutaneous nerve), which gave C6-T1 ventral rami contributions to the median nerve (Figure 3).

There was a typical branching pattern for C7, C8, and T1. The C7 ventral ramus continued as the middle trunk and then split into an anterior and posterior division. The C8 and T1 ventral rami formed the inferior trunk, which split into an anterior and posterior division. The anterior divisions of the middle and inferior trunks formed the medial cord, a branch of which contributed to the median nerve. The ulnar nerve branched from the medial cord as expected.

**Figure 4:**
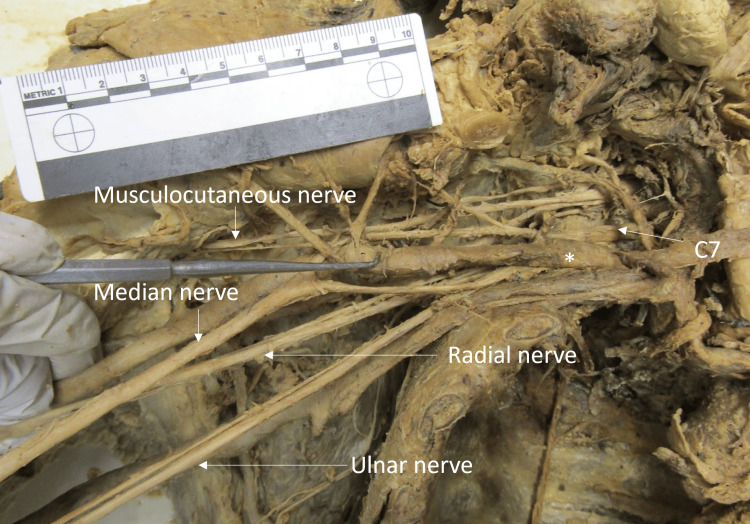
Right brachial plexus The right brachial plexus in its entirety, including the C5-C7 ventral rami, divisions, cords, and terminal branches. C7, C8, and T1 ventral rami branched typically, but the posterior cord received contributions from C5 and C6 individually rather than from medial and lateral cords. The subclavian artery is labeled with an asterisk (*). The ruler in the image measures 10 cm.

The posterior cord received contributions from all rami. The posterior cord typically receives the posterior divisions from the superior, middle, and inferior trunks. On this individual, posterior divisions arose from the middle and inferior trunks, as expected. However, due to the failure of the formation of the superior trunk, both the C5 and C6 individually contributed posterior divisions to the posterior cord. The axillary and radial nerves branched as expected from the posterior cord (Figure 4).

**Figure 5:**
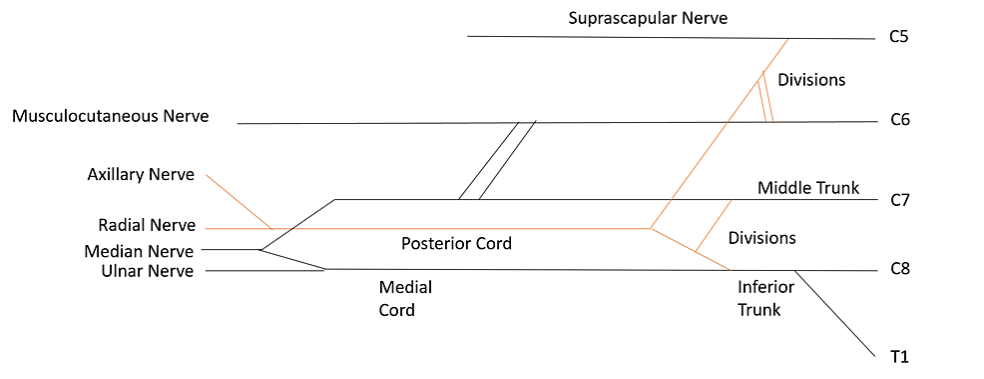
Brachial plexus schematic The schematic depicts the branching pattern exhibited by the described brachial plexus. The orange lines indicate a posterior position relative to the structures indicated by the black lines. The plexus did not converge into a superior trunk, nor did it converge into a lateral cord.

The schematic below depicts the plexus in its entirety (Figure 5), including the lack of a superior trunk and lateral cord and the typical branching pattern of the middle and inferior trunks and posterior and medial cords (Figure 4).

## Discussion

There are many well-described variations upon the typical brachial plexus branching pattern. Many such cases include the fusion of branches to make fewer than three cords. There have been multiple instances of the superior trunk fusing with C7-T1 to form one cord. In these cases, the terminal branches then divide from the single cord. This seems to be a fairly typical anomaly [1]. In another instance, the lateral cord pierced the coracobrachialis muscle, and the lateral root of the median nerve branched below the attachment of the coracobrachialis on the scapula [2]. There are also cases in which the median nerve is only derived from the medial cord rather than from the medial and lateral cords [2]. Finally, there are several cases in which the brachial plexus receives little to no input from T1 but has contributions from C4 (so-called ‘pre-fixed’) (25.5% of cases investigated) or receives little to no input from C5 but has contributions from T2 (‘post-fixed’) (2.5% of cases investigated) [4].

This donor differs from these ‘typical’ variations, in that there is a lack of convergence into a superior trunk and a lateral cord. Where most anomalies describe a plexus that converges to two or fewer trunks or cords, the brachial plexus in this discussion does not converge into a superior trunk and instead directly branches into anterior and posterior divisions. Additionally, there was no formation of a lateral cord, rather the suprascapular nerve arose only from the C5 ventral ramus and the musculocutaneous nerve arose only from the C6 ventral ramus. The median nerve thus received contributions from the medial cord and only small branches from the anterior division of C6 rather than from a lateral cord. These abnormalities can be attributed to variations in embryological development, and have implications for various surgical and anesthetic procedures.

Embryological basis and clinical implications

During development, the brachial plexus emerges from one axon extending from the spine into the arm bud. The environment in which this occurs has various spatial constraints and obstacles that are also developing such as blood vessels, the skeleton, and cartilage. These obstacles help guide the development of the brachial plexus as is seen in adult anatomy. Thus, variations in the branching pattern of the brachial plexus can often be attributed to variations in these anatomical spatial constraints [5]. For example, the lateral cord may split as it develops around veins. Some of these splits are due to how the subclavian artery develops from the intersegmental artery. If it develops above or below its typical seventh intersegmental level, this may disrupt brachial plexus development. Most anomalies of the brachial plexus can be explained by variations in blood vessel anatomy, and other variations are poorly understood [5]. However, these variations are exceedingly relevant in areas such as anesthesia, neurosurgery, and orthopedic surgery.

The late 1800s marked the dawn of a new era in pain management with the advent of anesthetic nerve blocks. William Halsted and Richard Hall conducted clinical trials in 1884, testing the efficacy of injecting 4% solutions of cocaine into the brachial plexus and tibial nerve to explore the concept of regional anesthesia and nerve blocks for procedures involving the upper and lower limbs [12]. To achieve regional anesthesia of the upper limb, the brachial plexus can be blocked at different locations along the trunks, divisions, cords, and terminal branches, depending on the area of interest [13]. Compared to general anesthesia, brachial plexus block (BPB) offers several advantages that make it a preferred alternative. These benefits include enhanced pain management, shorter hospitalization periods, and decreased incidence of systemic effects [14].

Understanding the variations in the formation of the brachial plexus holds immense value for various medical professionals, including anatomists, radiologists, anesthesiologists, neurosurgeons, vascular surgeons, and orthopedic surgeons. This knowledge plays a crucial role in guiding nerve block procedures, surgical approaches for tumors in the brachial plexus region, and orthopedic treatment of cervical rib injuries and other upper limb conditions. For example, neurosurgeons can leverage this understanding to enhance surgical interventions for nerve sheath tumors like schwannomas and neurofibromas [7,15]. Anesthesiologists and surgeons also benefit significantly from this knowledge, as it improves their guidance during nerve block procedures and surgical interventions. Furthermore, the historical context reveals that the advent of brachial plexus blocks in the late 1800s revolutionized pain management, providing advantages such as improved pain control, shorter hospitalization periods, and reduced systemic effects compared to general anesthesia [12,14]. Therefore, a comprehensive understanding of both the normal and abnormal formation of the brachial plexus contributes to better patient care and treatment outcomes across multiple medical disciplines.

## Conclusions

During a standard prosection of a 74-year-old male anatomical donor, anomalies in the brachial plexus branching pattern were observed, with the right brachial plexus displaying variations in the superior trunk and lateral cord. A number of analogous studies have been conducted, all of which reinforce that the prevalence of anomalies in the brachial plexus anatomy is considerably widespread. Addressing these anatomical irregularities is crucial for regional anesthesia, neurosurgical, and orthopedic procedures. Regional anesthesia offers a more efficient alternative to general anesthesia for upper limb and cervical procedures involving the brachial plexus. Prior verification of the brachial plexus nerves using nerve stimulation or ultrasound, along with the implementation of ultrasound guidance during brachial plexus blocks and other such procedures, can significantly improve the efficacy and patient-specific outcomes while minimizing the associated risks. These insights highlight the need for ongoing research and clinical vigilance to optimize the management of brachial plexus variations and enhance patient care in relevant medical specialties.
